# Merge-Generability as the Key Concept of Human Language: Evidence From Neuroscience

**DOI:** 10.3389/fpsyg.2019.02673

**Published:** 2019-11-29

**Authors:** Kyohei Tanaka, Isso Nakamura, Shinri Ohta, Naoki Fukui, Mihoko Zushi, Hiroki Narita, Kuniyoshi L. Sakai

**Affiliations:** ^1^Department of Basic Science, Graduate School of Arts and Sciences, The University of Tokyo, Tokyo, Japan; ^2^Department of English Language and Literature, Faculty of Letters, The University of Tokyo, Tokyo, Japan; ^3^Department of Linguistics, Faculty of Humanities, Kyushu University, Fukuoka, Japan; ^4^Graduate School of Languages and Linguistics, Sophia University, Tokyo, Japan; ^5^Faculty of Foreign Languages, Kanagawa University, Yokohama, Japan; ^6^Department of English, Faculty of Letters, Tokai University, Hiratsuka, Japan

**Keywords:** syntax, Chomsky Hierarchy, Merge, Merge-generability, inferior frontal gyrus, lateral premotor cortex, fMRI

## Abstract

Ever since the inception of generative linguistics, various dependency patterns have been widely discussed in the literature, particularly as they pertain to the hierarchy based on “weak generation” – the so-called Chomsky Hierarchy. However, humans can make any possible dependency patterns by using artificial means on a sequence of symbols (e.g., computer programing). The differences between sentences in human language and general symbol sequences have been routinely observed, but the question as to *why* such differences exist has barely been raised. Here, we address this problem and propose a theoretical explanation in terms of a new concept of “Merge-generability,” that is, whether the structural basis for a given dependency is provided by the fundamental operation Merge. In our functional magnetic resonance imaging (fMRI) study, we tested the judgments of noun phrase (NP)-predicate (Pred) pairings in sentences of Japanese, an SOV language that allows *natural*, unbounded nesting configurations. We further introduced two pseudo-adverbs, which *artificially* force dependencies that do *not* conform to structures generated by Merge, i.e., non-Merge-generable; these adverbs enable us to manipulate Merge-generability (*Natural* or *Artificial*). By employing this novel paradigm, we obtained the following results. Firstly, the behavioral data clearly showed that an NP-Pred matching task became more demanding under the Artificial conditions than under the Natural conditions, reflecting cognitive loads that could be covaried with the increased number of words. Secondly, localized activation in the left frontal cortex, as well as in the left middle temporal gyrus and angular gyrus, was observed for the [Natural – Artificial] contrast, indicating specialization of these left regions in syntactic processing. Any activation due to task difficulty was completely excluded from activations in these regions, because the Natural conditions were always easier than the Artificial ones. And finally, the [Artificial – Natural] contrast resulted in the dorsal portion of the left frontal cortex, together with wide-spread regions required for general cognitive demands. These results indicate that Merge-generable sentences are processed in these specific regions in contrast to non-Merge-generable sentences, demonstrating that Merge is indeed a fundamental operation, which comes into play especially under the Natural conditions.

## Introduction

The present study aims to support the concept of human language, by putting forth a new theoretical hypothesis and by providing novel experimental evidence drawn from neuroscience. Our newly designed functional magnetic resonance imaging (fMRI) experiment focused on the fundamental operation of human language – *Merge*, with its ramified functions in characterizing various formal dependencies and their computation in the brain. Merge is a simple and primitive combinatory operation that takes *n* objects (usually two, in the case of human language), say *X* and *Y*, and forms an unordered set of the objects (Chomsky, 1995, 2000). The literature of theoretical linguistics has converged on the hypothesis that human language at its core is a uniquely human system of unbounded Merge, and this simple operation is the single generative engine underlying the infinity of linguistic expressions.

If this hypothesis is correct, then it follows that natural linguistic dependencies (such as those defined over embedding and coordination) are possible only when phrase structures that lie behind the relevant dependencies are generable by unbounded Merge (Fukui, 2015). Capitalizing on the proposal put forth in Fukui (2015), we make the distinction between “Merge-generable” dependencies and “non-Merge-generable” dependencies. A dependency is Merge-generable if it is based on a structure generated by Merge; otherwise, the dependency is non-Merge-generable. The central role of Merge in characterizing linguistic dependencies, as explicitly depicted by the notion of Merge-generability just defined, leads to the following hypothesis:

(1) **Hypothesis:** Only Merge-generable dependencies are naturally computable as linguistic dependencies by the human language faculty.

This hypothesis makes sense under the “Merge-only” hypothesis above, because if there is no structure generated by Merge for a given dependency, there will be no strictly linguistic way to characterize such a dependency. Thus, Merge-generability sets a necessary condition for “linguistically possible” dependencies. Non-Merge-generable dependencies are, then, strictly speaking, “linguistically impossible.” This is a big – and crucial – line that we would like to draw between various types of dependencies defined over linguistic expressions.

Merge-generable dependencies (i.e., “linguistically possible” dependencies) are further divided into two subtypes. One subtype is a dependency that is based on a structure *totally* determined by Merge. This type of *totally* Merge-generable dependencies includes, among many other “core” dependencies, subject-predicate linking – typically instantiated by noun phrase (NP)-predicate (Pred) pairing – as observed in Japanese sentence-embedding (carried out by the so-called “External Merge;” see below), as well as filler-gap dependency and operator-variable relations in movement (created by the so-called “Internal Merge”). Note that the latter type of dependency is the one holding between more than one *copy* (occurrence) of the same, single syntactic object (Chomsky et al., 2019) (see also the “Discussion” section), and is thus different in nature from the former type of dependency that holds between distinct syntactic objects, NP and Pred for example. While much “processing/parsing” literature in psycholinguistics has been focused on filler-gap dependencies, we do not directly deal with this type of dependency between copies of the same syntactic object in this study, simply pointing out that filler-gap dependencies are totally Merge-generable.

The other subtype of Merge-generable dependency is the one such that although based on a structure generated by Merge (i.e., Merge-generable), the conditions for the relevant dependency are *not* totally determined by Merge alone; rather, it requires some other factors such as left-to-right precedence, isomorphy, and pragmatic factors. This subtype of dependencies is called *partially* Merge-generable, and it typically includes group reading and cross-serial interpretation in coordinate structures. Totally and partially Merge-generable dependencies are naturally expected to be treated differently in the brain, but the thorough and detailed experimental study of the different functioning of these dependencies falls outside of the scope of this article, and we leave the investigation of this important topic for future research, focusing on, in the present study, the crucial and fundamental distinction between Merge-generable and non-Merge generable dependencies.

Regarding the neural basis of Merge, in our previous fMRI study we demonstrated that Degree of Merger (DoM) accounted for syntax-selective activations in the pars opercularis and pars triangularis (L. F3op/F3t) of the left inferior frontal gyrus (L. IFG) (Ohta et al., 2013b). The DoM is the *maximum depth* of merged subtrees usually within an entire sentence, and it properly measures the complexity of tree structures. In contrast, the number of applications of (External) Merge in a sentence always becomes one less than the number of terminal nodes, *irrespective of sentence structures* (see Appendix S2 of Ohta et al., 2013b). The DoM domain, i.e., the subtrees where the DoM is calculated, is an entire sentence when there is no constraint, but this changes dynamically in accord with syntactic operations and/or task requirements (Ohta et al., 2013a). By comparing short postpositional phrases/sentences with word lists, another fMRI study also showed that Merge operations activated the L. F3op/F3t, as well as a smaller region in the posterior superior temporal sulcus (Zaccarella and Friederici, 2015). These fMRI studies strongly suggest that the fundamental structure-building operation, i.e., Merge, activates the L. F3op/F3t and the left lateral premotor cortex (L. LPMC), which have been proposed as grammar centers (Sakai, 2005). We are of course aware that there is a general methodological challenge, not disproportionately serious for the present study alone, but troublesome for any attempt to connect linguistic computation and neural activation: the problem as to how to substantively link cognition and neurobiology, as has been discussed in the literature (Chomsky, 2002; Embick and Poeppel, 2015). Our approach in this paper can be taken as an “integrated” approach in the sense of Embick and Poeppel (2015), with the goal of constructing an “explanatory” study in future work. We thus focus here on the above-mentioned main Hypothesis (1), and we report the findings revealed by our fMRI experiment that conforms to this overarching hypothesis.

**FIGURE 1:**
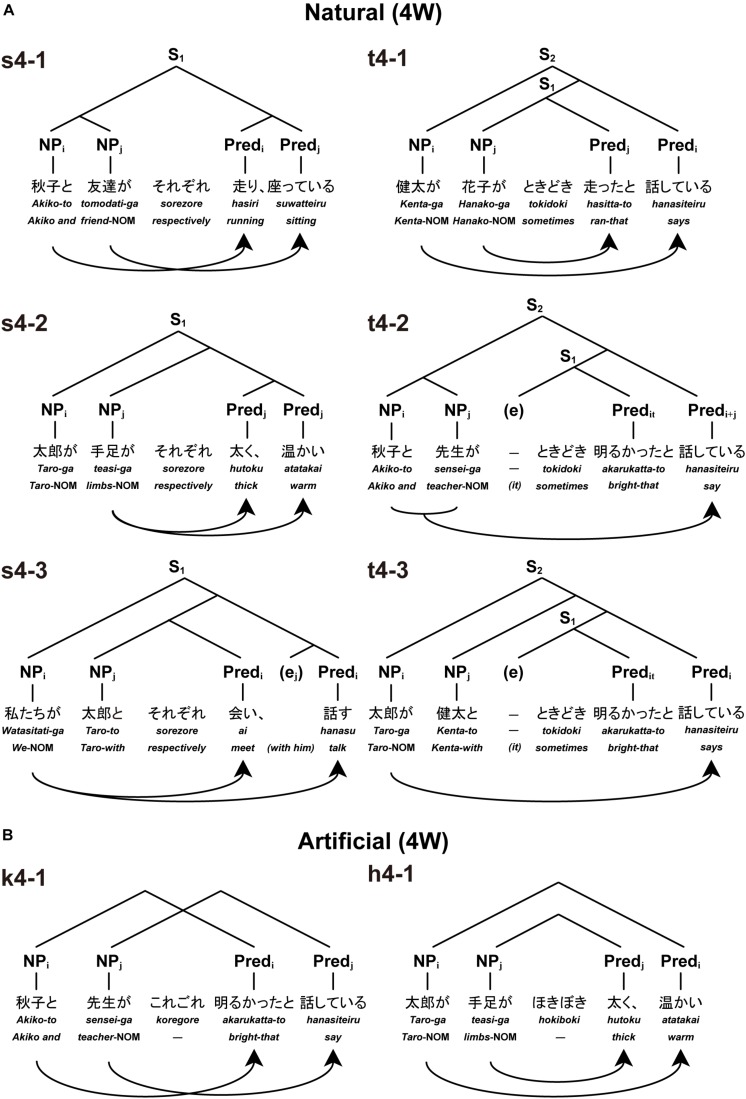
Basic types of Natural and Artificial sentences. We tested six sentence types each under Natural or Artificial conditions. Below each example in Japanese, phrases in Romaji and word-by-word translations in English are shown (NOM = a nominative case marker). Each type was presented as three visual stimuli in the order of noun phrases (NPs), an adverb, and predicates (Preds). The same subscript letters stand for structurally bound correspondences between an NP and a Pred in the sentence (S): e.g., NP_*i*_ and Pred_*i*_ indicate that these two elements are paired (Pred_it_ denotes the predicate of an indefinite subject “*it*”). Curved arrows also denote such NP-Pred pairings based on sentence structures. Each of tree structures represents unique structures for NPs and Preds. **(A)** There were six types of Natural sentences with four words. Left: three types of sentences with *sorezore*: e.g., “*Akiko and* [*her*] *friend are running and sitting, respectively*” (s4-1), “*As for Taro, [his] limbs are thick and warm, respectively*” (s4-2), and “*We meet with Taro and talk* [*with him*], *respectively*” (s4-3). Right: three types of sentences with *tokidoki*: e.g., “*Kenta says that Hanako sometimes ran*” (t4-1), “*Akiko and* [*her*] *teacher say that it was sometimes bright*” (t4-2), and “*Taro says with Kenta that it was sometimes bright*” (t4-3). **(B)** There were six types of Artificial sentences with four words, but only two of these are shown here. For the description of other four sentence types (k4-2, k4-3, h4-2, and h4-3), see the “Stimuli” section. Left: artificial cross-serial dependencies (pairing relations between NPs and Preds). Right: artificial nested dependencies. In these examples, pseudowords (“*koregore*” and “*hokiboki*”) artificially forced dependencies without conforming to Merge-generable structures.

We designed and conducted an fMRI experiment, the results of which provided a novel set of evidence supporting Hypothesis (1). As the target language, we chose Japanese because it exhibits unbounded nesting at the core of its syntax – sentence embedding. This is not the case in, say, English due to its SVO order. Japanese, by contrast, straightforwardly provides natural, unbounded nesting configurations, thanks to its SOV order. Natural sentences with various Merge-generable structures (Figure 1A) were first tested with native speakers of Japanese [the *Natural conditions*, using four-word (4W) and six-word (6W) sentences (excluding an adverb in the middle), i.e., Natural (4W) and Natural (6W), respectively]. On a separate day, we tested other conditions using two pseudo-adverbs (which do not exist in the actual Japanese), in which these dependencies switched with each other [the *Artificial conditions*, using 4W and 6W sentences, i.e., Artificial (4W) and Artificial (6W), respectively]. More specifically, these pseudowords were designed to require the participants to assign certain dependencies that do *not* conform to structures generated by Merge, i.e., non-Merge-generable. We predicted that Merge-generable dependencies would induce more specific activations in the grammar center and other syntax-related regions than non-Merge-generable dependencies would. By our testing of Merge-generability, we speculated that the fundamental status of Merge would be clearly elucidated, further highlighting the nature of the human language faculty.

### Theoretical Background

In this section, we explain the theoretical background of our study that is minimally necessary for understanding the significance of the experimental results reported in this paper.

One of the remarkable – and fundamental – discoveries of modern theoretical linguistics is the recognition that human language involves abstract “structures,” and that its mechanisms generate an infinite set of such structures. Linguistic expressions are not merely sequences of sounds or words; rather, they are associated with their “structural descriptions” – an array of abstract hierarchical structures – that determine their interpretations, both in terms of sound (pronunciation) and meaning (semantic interpretation). A speaker of a particular language has acquired and internalized a language in this sense – in current terminology, an “I-language” (Chomsky, 1986). The theory of an I-language is its generative grammar, a grammar of a particular language (henceforth in this text, “language” means “I-language”). A grammar is said to achieve *descriptive adequacy* (Chomsky, 1965), when it correctly describes the properties of the target language, i.e., how it generates a digitally infinite array of hierarchically organized abstract structural descriptions for linguistic expressions with the systematic interpretations at the two “interfaces” (sound and meaning) at which the language interacts with other internal systems. The general theory of languages is called Universal Grammar (UG), which is the theory of the genetic component of the language faculty that makes it possible for humans to acquire a language under limited conditions (Crain and Pietroski, 2001). UG determines the class of generative grammars that provide a set of correct structural descriptions for each language, thereby providing an explanation for the well-known facts about language acquisition (Berwick et al., 2011), in which case UG is said to achieve *explanatory adequacy*. It is important to note that in this conception of human language, there is virtually no room for the concept of “left-to-right” precedence or linear order, like how sounds or words are arranged left-to-right, without referring to hierarchical structures. Rather, it is always an array of abstract structures assigned to linguistic expressions – their structural descriptions – that plays a crucial role in the study of human language.

The relevant notion of *weak* and *strong* generation was introduced by Chomsky (1963, p. 325, 1965, p. 60), and the standard definition, adapted here from Chomsky (1965, p. 60), is as follows: “Let us say that a grammar *weakly generates* a set of sentences and that it *strongly generates* a set of structural descriptions…” Examples of weak generation are {aa, bb, aabb, …}, {John likes music, John ate an apple, …}, etc., depending on the Vocabulary of a grammar; those of strong generation are {[aa], [bb], [[aa] [bb]], …}, {[_S_ [_NP_ John][_VP_ [_V_ likes] [_NP_ music]]], [_S_ [_NP_ John] [_VP_ [_V_ ate] [_NP_[_Det_ an][_N_ apple]]]], …}, etc. Structural descriptions assigned by a grammar are complex objects and may be more than a single bracketing (or “tree”) structure (or a “layered” set structure, in the case of Merge systems), but rather, they can be a sequence of such abstract hierarchical structures (Chomsky, 1957, 1965). It should be clear, though, from the definition (and the examples provided) above that weak generation is just an enumeration of sentences (strings of elements), whereas strong generation has to do with more abstract hierarchical structures (or sequences of hierarchical structures) assigned to sentences by a grammar. On strong generation, an illuminating discussion is found in Kuroda (1976).

Theories of Merge aim to provide a minimal characterization of *strong generation*, namely the generation of structural descriptions of linguistic expressions (Chomsky, 1965). While theoretical linguistics has been developing increasingly better and refined accounts of strong generation, attention has been largely restricted in cognitive neuroscience to the study of *weak generation*, i.e., the formation of terminal, left-to-right strings of words (or word-like elements). There is a practical reason for the status of cognitive neuroscience in which a consistent focus has been placed on weak generation, virtually ignoring strong generation; it is much easier and more straightforward to deal with weak generation than strong generation, because you can literally *see* and readily construct the word-string stimuli for online experiments, while abstract hierarchical structures associated with the strings await in-depth theoretical investigations. Further, the research trend toward weak generation is boosted by a well-known result from formal language theory/automata theory, namely that the weak generative capacity of human language lies somewhere *above* the context-free phrase-structure grammar in the so-called “Chomsky Hierarchy” (Chomsky, 1959, 1963; Joshi, 1985).

The formal properties that have been highlighted and widely discussed in the literature are nested dependencies and cross-serial dependencies. Briefly put, nested dependencies are dependencies that hold between *x*_*i*_ and *y*_*i*_ (i.e., *x* and *y* with the same subscript) in the configuration *x*_1_*x*_2_ … *x*_*n*__–__1_*x_*n*_ … y_*n*_y_*n*_*_–__1_ … *y*_2_*y*_1_, forming a “nested” dependency structure, while cross-serial dependencies are dependencies holding between *x*_*i*_ and *y*_*i*_ in the configuration *x*_1_*x*_2_ … *x*_*n*_ … *y*_1_*y*_2_ … *y*_*n*_, forming a “crossing” dependency schema. And it has been observed that human language exhibits nested dependencies in a great number of instances, while it also shows, *in very limited contexts*, cross-serial dependencies. Based on this difference, it has been argued that the generative power of human language is beyond the bounds of finite-state grammar and is beyond the scope of context-free phrase-structure grammar, but perhaps stays, in terms of its weak generation, within the bounds of a certain class of context-sensitive phrase-structure grammar (Joshi, 1985). This claim makes sense only insofar as we restrict our attention to weak generation, but recall that, as we pointed out above, the nature of human language has to do with strong generation – the generation of structural descriptions of linguistic expressions. If this is true, then the whole discussion about weak generative capacities of various generative systems (grammars) may in fact be beside the point, as far as the empirical inquiry into the nature of human language is concerned. Questions of real empirical interest arise only when strong generation is at stake, or more importantly, the problem of “explanatory adequacy” (see above) is in focus, a matter that goes beyond even strong generation (Chomsky, 1963, 1965).

In fact, if we shift our attention from dependencies defined on terminal strings to how abstract hierarchical structures behind them are formed by linguistic computations, the well-known fact mentioned in the preceding paragraph concerning mysterious distribution of the two types of dependencies in human language can be rightly addressed. Consider, for example, the subject-predicate pairing in languages such as Japanese, as in the upper row in sentence (2) below, resulting from the “SOV” (Subject-Object-Verb/Predicate) word order (see the topmost right panel of Figure 1A for a real stimulus of nested configurations).

(2) [*Taro*-*ga*_1_ [*Hanako*-*ga*_2_ [*Ziro*-*ga*_3_ *odotta*_3_-*to*] *hanasita*_2_-*to*] *omotteiru*_1_]

[*Taro*-NOM [*Hanako*-NOM [*Ziro*-NOM *danced*-*that*] *said*-*that*] *thinks*] (NOM, a nominative case marker)

“*Taro*_1_ *thinks*_1_ *that Hanako*_2_ *said*_2_ *that Ziro*_3_ *danced*_3_”

In this structure, which is a typical sentence embedding configuration in Japanese, the only possible linking pattern is to associate a subject with its corresponding predicate in the manner indicated by the subscript numbers (the same number for each subject-predicate pair), forming nested dependencies generated by iterative applications of Merge. The other linking patterns, including cross-serial dependencies, are simply impossible. For example, sentence (2) can never mean that *Ziro thinks that Hanako said that Taro danced*. Even though this interpretation is plausible either semantically or pragmatically, it is just not a possible interpretation provided by the grammar of Japanese. By contrast, the nested dependencies as exemplified by sentence (2) are readily – and widely – available in other human languages as well.

On the other hand, cross-serial dependency, which is generally argued to be one of the characteristic properties that require more powerful *context-sensitivity*, is only available in very limited contexts, as has been widely acknowledged in the linguistic literature (see above). One representative case is the “respectively” reading of coordination (Bar-Hillel and Shamir, 1960). Consider the following example from Japanese.

(3) [[*Taro*_1_-*to Hanako*_2_-*to Ziro*_3_]-*ga* (*sorezore*) [*odori*_1_, *hanasi*_2_, *omotteiru*_3_]]

[[*Taro and Hanako and Ziro*]-NOM (*respectively*) [*dancing*, *saying*, *thinking*]]

“*Taro*_1_, *Hanako*_2_, *and Ziro*_3_ *are dancing*_1_, *saying*_2_, *and thinking*_3_, *respectively*”

The subject-predicate pairings in the Japanese sentence (3) (or its English counterpart for that matter) can be seen as exemplars of cross-serial dependencies (see the topmost left panel of Figure 1A). If the adverb *sorezore* “respectively” is absent, the so-called “group reading” is also possible, where the interpretation is such that *the group of people consisting of Taro, Hanako, and Ziro are collectively dancing, saying, and thinking*. However, other dependency patterns are impossible to obtain here. Thus, the specific question that should be addressed based on these facts about linking patterns exemplified in sentence (2) and (3) is as follows. Why is it that in a configuration such as sentence (2), only nested dependencies are allowed, whereas in sentence (3), cross-serial dependencies as well as group reading are allowed, with nested dependencies being mysteriously excluded? Note incidentally that context-sensitive phrase-structure grammar easily generates all kinds of dependencies in these cases, including non-existent cross-serial dependency for sentence (2), and also non-existent nested dependency for sentence (3). Thus, it fails to distinguish “linguistically possible” dependencies from “linguistically impossible” ones. As we will discuss in detail later on in the Discussion, the problem just mentioned can be appropriately addressed and naturally resolved only insofar as abstract structures generated by Merge are seriously taken into account.

It is simply impossible to tackle the problem just mentioned if we only look at terminal strings, because the examples (2) and (3) represent the same sequential patterns, with three NPs on the left and three Vs (or Preds) on the right (NP NP NP … V V V; see the Stimuli section for relevant discussion). However, if we turn our attention to the *structures* of these sentences, a clear picture emerges. To see this, let us consider first the availability of the nested dependencies, which is available in a sentence embedding structure such as that of sentence (2) but never possible in a coordinate structure such as that of sentence (3). We argue that the nested dependencies between NPs and Vs in sentence (2) are straightforwardly obtained as a consequence of iterative applications of Merge, as it combines an NP and a V, going on to embed a sentence within another sentence, as illustrated roughly in (4). Note that we abstract away from all the technical details of clausal architecture that are not directly relevant for our present discussion. In particular, in order to avoid unnecessary complications in illustration, we refrain from depicting the “functional” portions of a clause structure. Those “functional heads” such as T(ense) and C(omplementizer) are – if they are indeed syntactic functional heads in Japanese, not an innocent assumption – undoubtedly incorporated into the central clausal structure by Merge. And to the extent that they are incorporated by Merge, their existence does not affect our discussion. Thus, for simplicity, we omitted them in our exposition below. Also, for the gloss and translation, see (2).

(4) a. Merge(*Ziro*-*ga*_1_, *odotta*_1_-(*to*))

= {*Ziro*-*ga*_1_, *odotta*_1_-(*to*)}

– A verb phrase V(P)_1_ and NP_1_ are combined by Merge, forming a sentence S_1_

b. Merge({*Ziro*-*ga*_1_, *odotta*_1_-(*to*)}, *hanasita*_2_-(*to*))

= {{*Ziro-ga*_1_, *odotta*_1_-(*to*)}, *hanasita*_2_-(*to*)}

– S_1_ and V_2_ are combined by Merge, forming a V(P)_2_

c. Merge(*Hanako*-*ga*_2_, {{*Ziro*-*ga*_1_, *odotta*_1_-(*to*)}, *hanasita*_2_-(*to*)})

= {*Hanako*-*ga*_2_, {{*Ziro*-*ga*_1_, *odotta*_1_-(*to*)}, *hanasita*_2_-(*to*)}}

– V(P)_2_ and NP_2_ are combined by Merge, forming an S_2_

d. Merge({*Hanako*-*ga*_2_, {{*Ziro*-*ga*_1_, *odotta*_1_-(*to*)}, *hanasita*_2_-(*to*)}}, *omotteiru*_3_)

= {{*Hanako*-*ga*_2_, {{*Ziro*-*ga*_1_, *odotta*_1_-(*to*)}, *hanasita*_2_-(*to*)}}, *omotteiru*_3_}

– S_2_ and V_3_ are combined by Merge, forming a V(P)_3_

e. Merge(*Taro*-*ga*_3_, {{*Hanako*-*ga*_2_, {{*Ziro*-*ga*_1_, *odotta*_1_-(*to*)}, *hanasita*_2_-(*to*)}}, *omotteiru*_3_})

= {*Taro*-*ga*_3_, {{*Hanako*-*ga*_2_, {{*Ziro*-*ga*_1_, *odotta*_1_-(*to*)}, *hanasita*_2_-(*to*)}}, *omotteiru*_3_}}

– V(P)_3_ and NP_3_ are combined by Merge, forming an S_3_.

Since, this process is just a normal mode of applying Merge *recursively* (phase-by-phase, “phase” being a technical notion indicating a restrictive domain for rule applications), we say that such nested dependencies are totally Merge-generable. It therefore comes as no surprise that nested dependencies as exemplified in a sentence embedding structure such as that of sentence (2) are widely available in human language. Notice incidentally that the structures for the other linking patterns pointed out above in connection with example (2) cannot be generated by Merge in the way designated in (4), and thus are non-Merge-generable, which accounts for the unavailability of their associated interpretations.

Let us next consider the possibility of cross-serial dependencies between NPs and Vs in sentence (3). The crucial difference between sentences (2) and (3) in structure is that in the former sentence, neither the sequence of the NPs (*Taro*, *Hanako*, and *Ziro*) nor that of the Vs (*odotta*, *hanasita*, and *omotteiru*) form a *constituent* – a word or a group of words that function(s) as a single syntactic unit (i.e., a set) within a hierarchical structure, whereas in the latter sentence, the sequence of conjoined NPs or that of Vs each forms a constituent. A step-by-step derivation for sentence (3) is illustrated in (5) [see (3) for the gloss and translation].

(5) a. Merge(*Hanako*-*to*_2_, *Ziro*_3_-(*ga*))

= {*Hanako*-*to*_2_, *Ziro*_3_-(*ga*)}

b. Merge(*Taro*-*to*_1_, {*Hanako*_2_-(*to*), *Ziro*_3_-(*ga*)})

= {*Taro*-*to*_1_, {*Hanako*_2_-(*to*), *Ziro*_3_-(*ga*)}}

– NP_1_, NP_2_, and NP_3_ are combined by iterative Merge.

c. Merge(*hanasi*_2_, *omotteiru*_3_)

= {*hanasi*_2_, *omotteiru*_3_}

d. Merge(*odori*_1_, {*hanasi*_2_, *omotteiru*_3_})

= {*odori*_1_, {*hanasi*_2_, *omotteiru*_3_}}

– V_1_, V_2_, and V_3_ are combined by iterative Merge.

e. Merge({*Taro*-*to*_1_, {*Hanako*_2_-(*to*), *Ziro*_3_-(*ga*)}}, {*odori*_1_, {*hanasi*_2_, *omotteiru*_3_}})

= {{*Taro*-*to*_1_, {*Hanako*_2_-(*to*), *Ziro*_3_-(*ga*)}}, {*odori*_1_, {*hanasi*_2_, *omotteiru*_3_}}}

As shown in (5), the sequence of conjoined NPs and that of Vs in sentence (3) each forms a constituent (a set). This provides the grammatical basis for the group reading, which requires matching of the sequence of NPs and that of Vs as a whole. Thus, such a reading becomes readily available. In addition to this natural group reading, the cross-serial dependencies are also possible here. Merge forms the two constituents – the conjoined NPs and complex of Vs – and the interpretive mechanisms at the conceptual/thought interface apply in accord with an “isomorphy” condition which incorporates an insight of the “copying transformation” of Chomsky (1957) that requires parallel (isomorphic) hierarchical structures for the two constituents at hand, yielding the cross-serial interpretation. Thus, details of interpretive processes aside, it is clear that Merge sets out a necessary structural basis for cross-serial dependencies.

Needless to say, nested dependencies and other linking patterns are impossible in sentence (3), simply because Merge, as it applies to generate the structure of sentence (3), does *not* yield the structural basis for such dependencies and there is no other way to obtain these linking patterns. By contrast, in sentence-embedding constructions like sentence (2), neither the sequence of NPs nor that of Vs forms a constituent, and thus the group reading is impossible. Nor is the cross-serial dependency allowed in sentence (2), since there is no structural basis, i.e., constituency of the relevant elements, for such a dependency.

Thus, the generalization we can draw from these facts is that cross-serial dependencies in human language become possible only when the relevant terminal elements form a constituent. As we demonstrated above, Merge sets out the necessary structural condition, forming the relevant constituents in the coordinate structures such as that of sentence (3). However, Merge does *not* in and of itself provide a direct structural basis for cross-serial dependencies. This is probably why the interpretation requires a special device such as “*sorezore*” or “*respectively*” that effectively forces this interpretation, rather than the more natural (and apparently default) group reading, which is available only by virtue of Merge.

Summing up the discussion in this section, we have re-iterated the fundamental discovery of modern theoretical linguistics according to which the nature of human language critically depends on its mechanisms, particularly Merge – the basic and fundamental operation of (unordered) set-formation in syntax. Correspondingly, well-known results in formal language theory concerning the generation of dependencies defined over terminal strings (e.g., context-free vs. context-sensitive phrase-structure grammars) and the related discussion should be reconsidered and re-evaluated from the new theoretical point of view based on Merge. We have looked at two typical examples from Japanese, and we have suggested that these simple examples demonstrate important points about the nature of formal dependencies in human language. These points strongly suggest that dependencies are possible in human language only to the extent that they result from abstract structures generated by Merge, leading to the conclusion that it is Merge-generability that determines the availability of various dependencies in human language [Hypothesis (1)]. We will thus argue that in human language, the apparent generation of various “types” of dependency defined on terminal strings is rather illusory, emerging only as an epiphenomenon of linguistic computation.

## Materials and Methods

### Participants

We recruited 25 native speakers of Japanese. They were undergraduate students who had not majored in linguistics or language sciences. Two participants were dropped from our analyses due to their health conditions. We also dropped four participants, who showed larger head movements (see below) in ≥75% of runs under one or more of the four conditions [Natural (4W), Natural (6W), Artificial (4W), and Artificial (6W)]. We excluded three more participants, whose accuracy on one or more sentence types (see Figure 1) was ≤60% (the chance level was at most 34% as shown below). The remaining 16 participants [six females; mean ± standard deviation (SD) age: 20.1 ± 1.1 years] showed right-handedness (laterality quotients: 81 ± 10) as determined by the Edinburgh inventory (Oldfield, 1971). None had a history of neurological or psychiatric diseases. Prior to participation in the study, written informed consent was obtained from each participant after the nature and possible consequences of the study were explained. Approval for the experiments was obtained from the Institutional Review Board of The University of Tokyo, Komaba Campus.

### Stimuli

As visual stimuli, we first prepared sentences under the Natural conditions, which were all grammatical and meaningful in Japanese. Under the Natural (4W) condition, there were 30 sentences in each of *six* sentence types (s4-1, s4-2, s4-3, t4-1, t4-2, and t4-3; see Figure 1A). Every sentence with four words (excluding an adverb) had two noun phrases (NPs, subjects), an adverb, and two predicates (Preds) in the form of NP-NP-Adverb-Pred-Pred. Under the Natural (6W) condition, there were 30 sentences in each of six sentence types (s6-1, s6-2, s6-3, t6-1, t6-2, and t6-3; see Supplementary Figure 1). Every sentence with six words (excluding an adverb) was in the form of NP-NP-NP-Adverb-Pred-Pred-Pred. Since Japanese lacks overt, semantics-free subject-predicate formal agreement, we chose another phenomenon in the language, namely, the subject-predicate linking, which in fact has been often utilized in the formal language/automata theory literature, and which, like most other linguistic dependencies, may not be immune from semantic, pragmatic, and other factors. We carefully examined the relevant phenomena to see if the nature of linking patterns is actually independent from those factors [*cf*. our discussion about example (2) above], and, as we will present below, we paid close attention to controlling non-syntactic factors as much as possible in our experiments.

For the nouns, we used common names of persons (e.g., “*Taro*”), (singular) animate nouns [e.g., “*sensei*” (teacher)], their plural forms [e.g., “*sensei-gata*” (teachers)], and part(s) of body [e.g., “*teasi*” (limbs)]. For the predicates, we used transitive verbs [e.g., “*kangae-ru*” (think)] (all of these select a clausal complement), intransitive verbs [e.g., “*odor-u*” (dance)], and adjectives [e.g., “*akaru-i*” (bright)]. Adjectives in Japanese act as Preds without copula verbs, and they have their own present and past forms {e.g., “*akaru-i*” [(is) bright]; “*akaru-k-atta*” [(was) bright]}. To avoid the undesirable possibility of default group-reading (which collectively relates all NPs to all Preds as a group) for the s4-1 (see Figure 1A) and s6-1 types (see Supplementary Figure 1), we selected at least two verbs indicating actions that cannot be collectively performed at the same time [e.g., “*hasiri*” (running) and “*suwatteiru*” (sitting)]. For these types, we also put a last predicate in progressive form, which was the case for all sentences with *tokidoki* (see below).

The adverbs under the Natural conditions were either “*sorezore*” (*respectively*; denoted here as “s” for such types) or “*tokidoki*” (*sometimes*; denoted here as “t” for such types), which were presented in *hiragana* (the basic Japanese syllabary that represents each mora in the Japanese language). While nested dependencies are created in sentence embedding constructions with or without an adverb, cross-serial dependencies are created only with the help of *sorezore* in coordinate configurations, in a way similar to English sentences with *respectively*: e.g., *Taro and Hanako sang and danced, respectively*. Note that the Merge-generable syntactic structures are naturally generated under these conditions.

We used three types of grammatical particles, which represent canonical case markings and syntactic information in Japanese: the nominative case marker *-ga* (which is realized as -*wa* when the subject represents the topic of the sentence; thus, we used -*wa* for s6-2, t6-1, and t6-2), a postposition *-to* (*with/and*), and a complementizer *-to* (*that*). In the sentences with *tokidoki*, the complementizer was placed at the end of the first Pred under 4W (Figure 1A), and of the first and second Preds under 6W. Each subject-predicate pair could not be made correctly, if rather rare and non-canonical usages of -*ga* – such as object marking and an external possessor – were employed. To correctly make each subject-predicate pair, the participants had to use *-ga* as a canonical nominative subject case marker. Since a Pred in past-tense form with a complementizer *-to* cannot be interpreted as a conditional clause like -*suru*-*to* containing a Pred in a present-tense form, we used, in an attempt to avoid the unwanted conditional clause interpretation, a past-tense form for all the Preds except for the last one in the sentences with *tokidoki*. In the sentences with *sorezore*, all the Preds except for the last one took an adverbial form, forming conjunctives for the Preds. In order to prevent participants from anticipating certain dependencies from particle patterns alone, we used NPs with the same particle patterns in two sentence types (with either *sorezore* or *tokidoki*) under 4W (e.g., an NP-*to-*NP-*ga*-Adverb-Pred-Pred pattern is used in s4-1 and t4-2). Due to syntactic characteristics of Japanese, this procedure was not possible under 6W.

Under the Artificial conditions, we used the same set of phrases as with the Natural conditions except that the adverbs *sorezore* and *tokidoki* were replaced with pseudo-adverbs, or phonotactically legal pseudowords, *koregore* (denoted here as “k”) and *hokiboki* (denoted here as “h”). There were 30 different sentences for each of six types of Artificial sentences (for two representative types, see Figure 1B; for six types with six words, see Supplementary Figure 2). Using six examples for each condition, we instructed the participants to pay attention to the fact that each pseudo-adverb determined correspondence among the other four or six words (see the Appendix of Supplementary Material). As shown in Figure 1B, nested or cross-serial dependency was enforced depending on which of these pseudo-adverbs was used. More specifically, the pseudo-adverb *koregore* artificially imposed *cross-serial* dependency (see k4-1 which is made from t4-2), as shown in example (6) below, in which brackets and indices denote artificial reading. This linking pattern is impossible as a real Japanese sentence. The pseudo-adverb *hokiboki*, on the other hand, artificially imposed *nested* dependency (see h4-1 which is made from s4-2), as illustrated by example (7) below. Again, the linking pattern is prohibited as an actual Japanese sentence. Both examples (6) and (7) thus *deviate* from Merge-generable structures.

(6) [[*Taro-ga*_i_ *inu-ga*_j_ *Hanako-ga*_k_] (*koregore*) [*aruita-to*_i_ *kizuita-to*_j_ *hanasiteiru*_k_]]

[[*Taro*-NOM *dog*-NOM *Hanako*-NOM] (–) [*walked-that noticed-that says*]]

“*Taro*_i_ *walks*_i_, *the dog*_j_ *notices*_j_, *and Hanako*_k_ *says*_k_”

(7) [*Taro-to*_i_ [*inu-to*_j_ [*Hanako-ga*_k_ (*hokiboki*) *kizuki*_k_,] *aruki*_j_,] *hanasiteiru*_i_]

[*Taro and* [*dog and* [*Hanako*-NOM (–) *noticing*,] *walking*,] *saying*]

“*Taro*_i_ *says*_i_, *the dog*_j_ *walks*_j_, *and Hanako*_k_ *notices*_k_”

Here, the same indexed letters indicate each NP-Pred pairing. These examples became thus ungrammatical, due to the illegitimate linking patterns imposed by the artificial adverbs.

Under the Artificial (4W) condition, we prepared *six* sentence types (k4-1, k4-2, k4-3, h4-1, h4-2, and h4-3, which were made from *four* sentence types under the Natural (4W) condition: [k4-1 and h4-2 from t4-2], [h4-1 and k4-2 from s4-2], [k4-3 from s4-3], and [h4-3 from t4-3]. Note that the original sentences with cross-serial and nested dependencies (i.e., s4-1 and t4-1, respectively) were not used under the Artificial (4W) condition, because they were conflicting with each other. Thus, the Artificial condition included two types of cross-serial and nested dependencies for the task, as well as four types derived from the original sentences (i.e., s4-2, s4-3, t4-2, and t4-3). The examples (6) and (7), in which nested and cross-serial dependencies were switched with each other, are presented above for the purpose of explanation only. The same procedures were used for the Artificial (6W) condition as well (see Supplementary Figure 2).

The resultant artificial NP-Pred pairings were all meaningful in terms of selectional restrictions on the words we used. We assessed the plausibility of the NP-Pred pairs (typical 20 pairs each for those used under the Natural conditions alone, Artificial conditions alone, or both), by asking their likelihood (Likert or five-point scale) to participants (*n* = 9), and the likelihood was not significantly different from the highest point (“definitely so”) under both Natural and Artificial conditions (one sample *t*-test, corrected *p* > 0.05). The NP-Pred pairs were hence equally plausible; non-syntactic factors such as semantic and pragmatic knowledge, as well as frequencies of constructions, were strictly controlled between the conditions. Merge-generable and non-Merge-generable dependencies were thus expected to be realized under the Natural and Artificial conditions, respectively. Accordingly, we tested the participants first under the Natural conditions, and then under the Artificial conditions to compare *Merge-generability* (Natural vs. Artificial).

Each sentence was serially presented in three groups of NPs, adverb, and Preds. Each of these phrases was shown with two to six yellow characters in *hiragana* and *kanji* (the adopted logographic Chinese characters used in written Japanese) for 2.5 s (4W) or 3.5 s (6W) with an interval of 0.2 s after each group. The participants were instructed to read each sentence including particles like -*wa*, *-ga*, or *-to*. After the presentation of the sentence, a “question-set” was presented, which contained one of the Preds in its upper row, as well as three (4W) or four (6W) NPs in its lower row. The NPs in a question-set were chosen from those contained in the sentence, together with a conjunction of two NPs with *-to*, or an NP which was *not* contained in the sentence; these possibilities were informed to the participants. In the question-set, the NPs were always presented without any particle, and the Pred in the present form in order to avoid the use of particles or verb forms as cues. Question-sets were presented for 2.0 s (4W) or 3.0 s (6W) with a post-trial interval of 1.9 s; each trial lasted for 12.0 s (4W) or 16.0 s (6W).

The stimuli were presented against a dark background at the center of an eyeglass-like MRI-compatible display (resolution, 800 × 600; VisuaStim XGA, Resonance Technology Inc., Northridge, CA, United States), and the participants wore earplugs. For fixation, a red cross was always displayed at the center of the display, and the participants were instructed to keep their eyes on this position. The stimulus presentation and the collection of behavioral data [accuracy and reaction times (RTs)] were controlled using Presentation software (Neurobehavioral Systems, Albany, CA, United States).

### Tasks

Under both Natural and Artificial conditions, we used a task of NP-Pred matching, in which the participants were instructed to note *all* of the two (4W) or three (6W) NP-Pred pairs based on dependencies. NP-Pred matching under the *Natural* conditions imposed building syntactic structures of a given sentence (see Figure 1), rather than “word-to-word correspondence,” and thus required syntactic judgment at the sentence level; the task cannot be solved correctly by semantic or pragmatic judgment (see above). The participants then chose one of the three (4W) or four (6W) NPs corresponding to the Pred on the question-set (see above), by pressing a button on a handheld controller (see the Appendix of Supplementary Material). After these instructions were given to the participants, the participants were trained on each condition (4W or 6W) outside the scanner, until they confidently performed the task for two consecutive runs (at least four correct trials out of six trials per run). The condition of 6W was always tested after that of 4W, with a short break. During the MR scanning, no feedback on each trial’s performance was given to any participant.

The sentences of six types were presented randomly in the same frequency. A single run of MR scans contained 19 (4W) or 13 (6W) trials of either task. For every participant, the task with eight runs for 4W and nine for 6W under the Natural conditions were first conducted, and then the task under the Artificial conditions were tested in another day. By separating the task trials under the Natural and Artificial conditions in this order, we imposed the participants to read sentences in a natural way while performing the task under the Natural conditions. On the other hand, the participants might perform the task with a strategy like puzzle-solving under the Artificial conditions.

### MRI Data Acquisition

For the MRI data acquisition, a participant was in a supine position, and his or her head was immobilized inside the radio-frequency coil. The MRI scans were conducted on a 3.0 T MRI system equipped with a bird-cage head coil (GE Signa HDxt 3.0T; GE Healthcare, Milwaukee, WI, United States). During the fMRI session, we scanned 30 axial 3-mm thick slices with a 0.5-mm gap, covering the volume range of –38.5 to 66 mm from the anterior to posterior commissure (AC-PC) line in the vertical direction, using a gradient-echo echo-planar imaging (EPI) sequence [repetition time (TR) = 2 s, echo time (TE) = 30 ms, flip angle (FA) = 90°, field of view (FOV) = 192 × 192 mm^2^, resolution = 3 × 3 mm^2^). In a single scan, we obtained 114 [Natural (4W) and Artificial (4W)] or 104 [Natural (6W) and Artificial (6W)] volumes, in which the first six or eight images (the first dummy trial in each scan), for the rise of the MR signals were discarded. High-resolution T1-weighted images of the whole brain (192 axial slices, 1.0 × 1.0 × 1.0 mm^3^) were acquired from all participants with a three-dimensional fast spoiled gradient recalled acquisition in the steady state (3D FSPGR) sequence (TR = 8.4 ms, TE = 2.6 ms, FA = 25°, FOV = 256 × 256 mm^2^). These structural images were used for normalizing the fMRI data.

### fMRI Data Analyses

The fMRI data were analyzed by using SPM12 statistical parametric mapping software (Wellcome Trust Centre for Neuroimaging)^1^ (Friston et al., 1995) implemented on MATLAB software (MathWorks, Natick, MA, United States). We confirmed that all available fMRI data were free from large head movements, with a translation of <3 mm in the three directions and with a rotation of <2° around the three axes. The acquisition timing of each slice was corrected using the middle slice (the 15th slice chronologically) as a reference for the fMRI data. The time-series data in multiple runs were then realigned to the first volume in all runs, and resliced using seventh-degree B-spline interpolation, so that each voxel of each functional image matched that of the first volume.

The T1-weighted structural image of each participant was aligned to the AC-PC line, and coregistered to the mean functional image generated during the realignment of the fMRI data. Each T1-weighted image was bias-corrected with light regularization, and segmented to the gray matter, white matter, cerebrospinal fluid, bone, other soft tissues, and air by using default tissue probability maps and a standard tool in the SPM12 that uses an affine regularization to warp images to the International Consortium for Brain Mapping East Asian brain template (Ashburner and Friston, 2005). The realigned functional images were also spatially normalized to the standard brain space as defined by the Montreal Neurological Institute (MNI), which converted voxel sizes to 3 × 3 × 3 mm^3^ and smoothed images with an isotropic Gaussian kernel of 9-mm full-width at half maximum.

In a first-level analysis (i.e., the fixed-effects analysis) for each participant, hemodynamic responses induced by the correct-response trials for each session were modeled with a boxcar function with a duration of 7.9 s (4W) or 10.9 s (6W) from the onset of each visual stimulus, i.e., the length of the time for the five/seven words, as well as with a duration of 2 s (4W) or 3 s (6W) from the onset of a question. The boxcar function was then convolved with a hemodynamic response function, and low-frequency noises were removed by high-pass filtering at 1/128 Hz. To minimize the effects of head movement, the six realignment parameters obtained from preprocessing were included as a nuisance factor in a general linear model. The images of the six conditions were then generated in the general linear model for each participant and used for our intersubject comparison in a second-level analysis (i.e., the random-effects analysis).

In the second-level analysis, we performed a repeated measures analysis of variance (rANOVA) with a *t*-test, the results of which were thresholded at uncorrected *p* < 0.001 for the voxel level, and at corrected *p* < 0.05 for the cluster level, with topological false discovery rate (FDR) correction across the whole brain (Chumbley and Friston, 2009). For the anatomical identification of activated regions, we used the Anatomical Automatic Labeling method^2^ (Tzourio-Mazoyer et al., 2002) and the labeled data as provided by Neuromorphometrics Inc.^3^, under academic subscription.

## Results

### Behavioral Data

**FIGURE 2:**
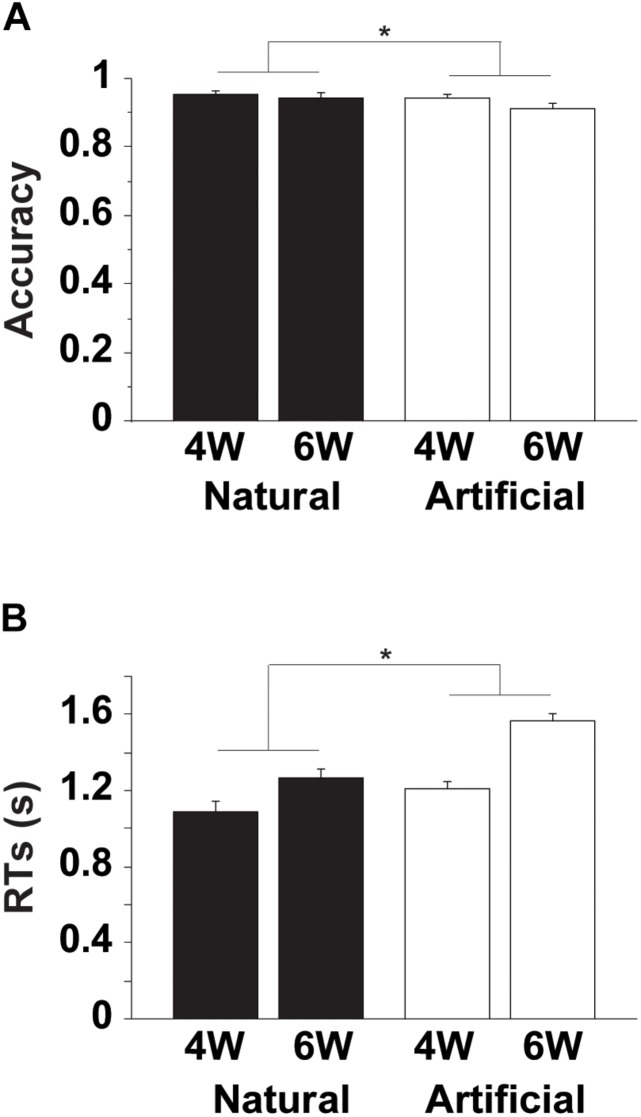
Behavioral data. **(A)** Accuracy for an NP-Pred matching task. Filled and open bars represent Natural and Artificial conditions, respectively, each with either 4W (two NPs and two predicates) or 6W (three NPs and three predicates) conditions. Error bars denote the standard error of the mean (SEM) for the participants. ^∗^Corrected *p* < 0.01. **(B)** Reaction times (RTs) from the onset of a question-set for judgment. Only correct trials were included for RTs.

The accuracy and RTs are shown in Figure 2. For the accuracy, an rANOVA with two factors [Merge-generability (Natural, Artificial) × word numbers (4W, 6W)] showed that both main effects of Merge-generability [*F*(1, 15) = 10, *p* = 0.006] and word numbers [*F*(1, 15) = 6.4, *p* = 0.02] were significant, without the interaction between them [*F*(1, 15) = 1.7, *p* = 0.2] (Figure 2A). The main effect of Merge-generability was due to lower accuracy under the Artificial conditions, while the main effect of word numbers may be simply caused by processing loads (see Supplementary Figures 1, 2).

Regarding RTs, there were significant main effects of Merge-generability [*F*(1, 15) = 26, *p* = 0.001] and word numbers [*F*(1, 15) = 119, *p* < 0.0001], as well as the significant interaction between them [*F*(1, 15) = 18, *p* < 0.001] (Figure 2B). In addition to consistent results with the accuracy, the significant interaction suggests that general cognitive loads under the Artificial conditions became more demanding for the increased number of words.

### Modulation of the Cortical Activation by Natural and Artificial Conditions

**FIGURE 3:**
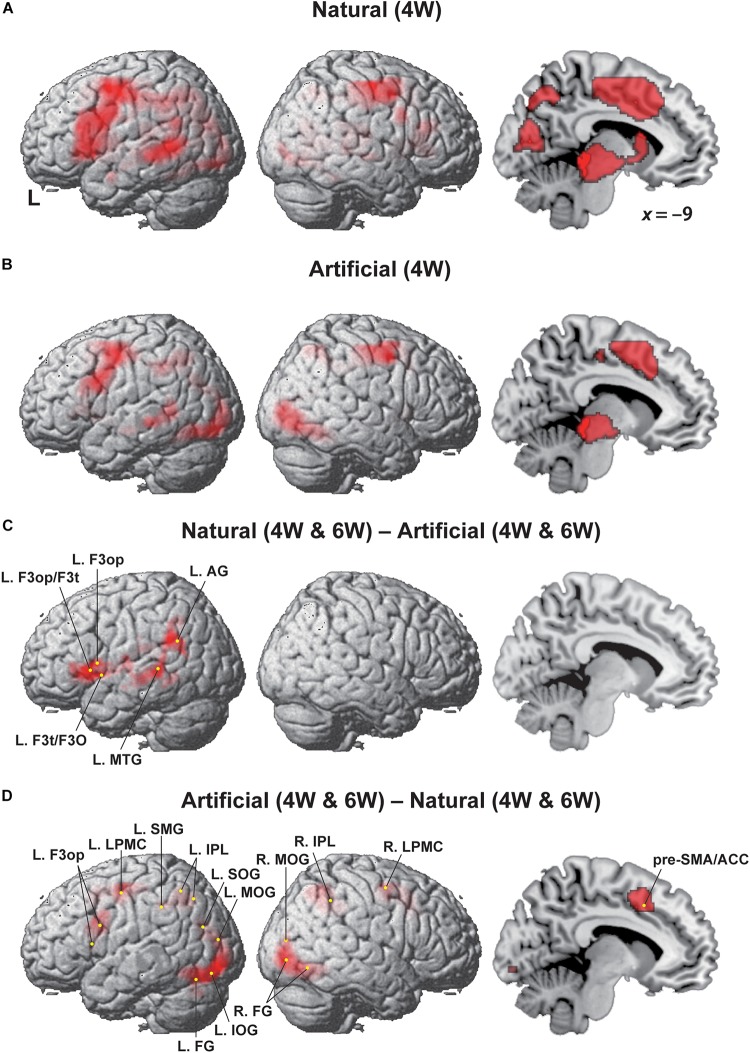
Modulation of the cortical activation by Natural and Artificial conditions. Regions identified by **(A)** Natural (4W), **(B)** Artificial (4W), **(C)** [Natural (4W and 6W) – Artificial (4W and 6W)], and **(D)** [Artificial (4W and 6W) – Natural (4W and 6W)]. Exclusive masks of [– Artificial (i.e., negative activation)] and [– Natural] (uncorrected *p* < 0.001) were applied to the comparisons of **C** and **D**, respectively. Activations were projected onto the left (L) and right lateral surfaces, and medial section (*x* = –9) of a standard brain (FDR-corrected *p* < 0.05). Each yellow dot indicates the local maxima of activated regions. See Table 1 for the stereotactic coordinates of activation foci.

As shown in Figure 3A, the most prominent activation under the Natural (4W) condition was mostly localized in the left frontal cortex, spanning most of the L. LPMC, L. F3op/F3t, and the orbital part of the inferior frontal gyrus (L. F3O), together with the left middle temporal gyrus (L. MTG). In addition to these language-related regions, additional activation was observed in the right hemisphere, such as the right LPMC (R. LPMC) and parietal cortex, together with medial regions including the pre-supplementary motor area (pre-SMA), anterior cingulate cortex (ACC), cuneus, caudate nucleus, and tegmentum/tectum. Under the Artificial (4W) condition, in contrast, the left frontal activation was greatly reduced to the dorsal portion (Figure 3B), and the left temporal activation was also decreased. The other right and medial regions were mostly consistent with those under the Natural (4W) condition. Regarding the 6W conditions, the overall activation patterns were similar to those under the 4W conditions, and left frontal activations were not enhanced as expected. Therefore, we combined the 4W and 6W conditions for subsequent analyses.

We then examined the [Natural (4W and 6W) – Artificial (4W and 6W)] contrast (Figure 3C), and found clearly localized activation in the ventral portion of the left frontal cortex, including the L. F3op, L. F3t, and L. F3O, as well as the L. MTG and the left angular gyrus (L. AG) (Table 1). On the other hand, the [Artificial (4W and 6W) – Natural (4W and 6W)] contrast resulted in a completely different pattern of activation (Figure 3D). As mentioned above, the left frontal activation was greatly reduced to the dorsal portion of the L. LPMC and L. F3op. Moreover, activated regions were more wide-spread in such regions as the R. LPMC, pre-SMA/ACC, left supramarginal gyrus (L. SMG), bilateral inferior parietal lobule (IPL), bilateral middle occipital gyrus (MOG), left superior/inferior occipital gyrus (L. SOG/IOG), and bilateral fusiform gyrus (FG). The pre-SMA/ACC activation was much stronger under the Artificial (6W) condition than under the Natural (6W) condition, although this tendency was reversed under the Natural (4W) and Artificial (4W) conditions. These results indicate that the ventral portion of the grammar center was critically activated under the Natural conditions, providing clear evidence that the Natural (Merge-generable) and Artificial (non-Merge-generable) sentences were differentially processed in the brain.

**TABLE 1:** Regions with enhanced activations under the Natural or Artificial condition.

**Brain regions**	**BA**	**Side**	***x***	***y***	***z***	***Z***	**Voxels**
**Natural (4W and 6W) – Artificial (4W and 6W)**							
F3op	44	L	−60	11	8	4.9	653
F3op/F3t	44/45	L	−54	14	5	4.8	^∗^
F3t/F3O	45/47	L	−42	8	−1	4.7	^∗^
MTG	21	L	−66	−34	2	4.6	^∗^
AG	39	L	−51	−52	26	5.8	^∗^
**Artificial (4W and 6W) – Natural (4W and 6W)**							
LPMC	6/8	L	−33	−7	47	4.9	785
		R	21	−7	53	4.6	^∗^
pre-SMA/ACC	6/8/32	M	−9	11	47	5.9	^∗^
		M	12	14	41	6.1	^∗^
		M	12	5	59	4.4	^∗^
LPMC	6/8	L	−54	8	35	4.3	165
F3op	44	L	−45	8	23	5.7	^∗^
		L	−33	14	8	3.9	^∗^
IPL	7/40	L	−27	−55	50	4.6	176
		L	−18	−67	47	4.1	^∗^
SMG	40	L	−36	−40	41	4.1	^∗^
IPL	7/40	R	27	−52	44	7.2	274
SOG	7/19	L	−27	−73	26	5.3	592
MOG	18/19	L	−27	−82	14	4.9	^∗^
IOG	18/19	L	−39	−79	−13	7.2	^∗^
FG	19	L	−42	−70	−16	6.8	^∗^
MOG	18/19	R	30	−85	14	4.9	417
FG	19	R	30	−85	−4	Inf	^∗^
		R	39	−67	−10	6.2	^∗^

*Stereotactic coordinates (*x*, *y*, *z*) in the MNI space (mm) are shown for each activation peak of *Z* values. The threshold was set at FDR-corrected *p* < 0.05 for the cluster level. BA, Brodmann’s area; L, left; R, right; M, medial; F3op/F3t/F3O = opercular/triangular/orbital part of the inferior frontal gyrus; LPMC, lateral premotor cortex; pre-SMA, pre-supplementary motor area; ACC, anterior cingulate cortex; AG, angular gyrus; SMG, supramarginal gyrus; IPL, inferior parietal lobule; MTG, middle temporal gyrus; FG, fusiform gyrus; SOG/MOG/IOG, superior/middle/inferior occipital gyrus. The region with an asterisk is included within the same cluster shown one row above.*

## Discussion

By employing a novel paradigm to manipulate Merge-generability (Natural or Artificial), we obtained the following three striking results. Firstly, the behavioral data clearly showed that the NP-Pred matching task became more demanding under the Artificial conditions than under the Natural conditions (Figure 2), reflecting cognitive loads that could be covaried with the increased number of words. Secondly, localized activation in the L. F3op, F3t, and F3O, as well as in the L. MTG and L. AG, was observed for the [Natural (4W and 6W) – Artificial (4W and 6W)] contrast (Figure 3C), indicating specialization of these left regions in syntactic processing. Any activation due to task difficulty was completely excluded from activations in these regions, because the Natural conditions were always easier than the Artificial ones (see Figure 2). And finally, the [Artificial (4W and 6W) – Natural (4W and 6W)] contrast resulted in the dorsal portion of the L. LPMC and L. F3op (Figure 3D), together with wide-spread regions required for general cognitive demands, such as visual attention (in the bilateral MOG and L. SOG/IOG), error detection (in the pre-SMA/ACC), and cognitive conflict (just as during a Stroop task) (Bush et al., 2000). These results indicate that Merge-generable sentences are processed in these specific regions in contrast to non-Merge-generable sentences, demonstrating that Merge is indeed a fundamental operation, which comes into play especially under the Natural conditions.

As explained in the Introduction, Merge is the fundamental local structure-building operation proposed by modern linguistics (Chomsky, 1995), which reflects a formal property of the competence system. Merge itself would be theoretically “costless,” requiring no driving force for its application (Saito and Fukui, 1998; Chomsky, 2004; Fukui, 2011; Chomsky et al., 2019). In addition to Merge, an indispensable operation in any language-like symbolic system, the DoM also seems to play a role in accounting for enhanced activation under the Merge-generable Natural conditions (Figure 3C); note that the DoM remained at a minimum (one) for *artificially* forced dependencies without conforming to Merge-generable structures (see Figure 1B). As we noted in the Introduction, further experimental studies are required to clarify whether totally Merge-generable (e.g., nested) and partially Merge-generable (e.g., cross-serial) dependencies are analyzed differently in the brain, i.e., in terms of differential sub-regions and/or activation levels.

Neuroimaging studies have established that syntactic processing selectively activates the L. F3op/F3t and L. LPMC (Stromswold et al., 1996; Dapretto and Bookheimer, 1999; Embick et al., 2000; Hashimoto and Sakai, 2002; Friederici et al., 2003; Musso et al., 2003), indicating that these regions have a critical role as grammar centers (Sakai, 2005). We also observed activations in the L. F3op/F3t and L. LPMC in our studies using sentences with non-canonical word orders, which contained filler-gap dependency and operator-variable relations in movement (created by the “Internal Merge”) (Kinno et al., 2008; Ohta et al., 2017; Tanaka et al., 2017). Moreover, our magnetoencephalography studies revealed a significant increase in the responses in the L. IFG, which reflected predictive effects on a verb caused by a preceding object in a short sentence (Iijima et al., 2009; Inubushi et al., 2012; Iijima and Sakai, 2014). In the present study, we observed selective activation in the L. F3op, L. F3t, and L. F3O in the [Natural (4W and 6W) – Artificial (4W and 6W)] contrast, which is consistent with these previous findings. Our present findings provide further and significant experimental evidence to support the hypothesis that the concept of Merge-generability plays a critical role in the processes subserved by the grammar centers.

Compared with the ventral portion of the grammar centers (i.e., the L. F3op, L. F3t, and L. F3O), the dorsal portion (the L. LPMC, or the left dorsal prefrontal cortex) has been shown to be involved in more automatic or implicit aspects of syntactic processing (Hashimoto and Sakai, 2002), while the R. LPMC was supportively required for syntactic processing (Kinno et al., 2014) or for memorizing mere strings (requiring memory span) (Ohta et al., 2013b). Moreover, the L. LPMC activations were particularly enhanced for scrambled, i.e., object-initial sentences (Kinno et al., 2008), which were also confirmed by lesion studies (Kinno et al., 2009, 2014). In the present study, L. LPMC activations were enhanced under the Artificial conditions (see Figure 3D), which required more pattern-based or procedural strategy – just as in the case of puzzle-solving – for artificially matching an NP-Pred pair. The left frontal activations were not enhanced as expected under the Natural (6W) condition in our experiment. This is probably because the task became more “mechanical,” requiring less conscious efforts and thus inducing less activations to process, as the number of words in the sentences increases.

It is instructive to note in this connection that while there has been much discussion on nested constructions/structures in the literature, there has been virtually no reference, as far as we are aware, to cross-serial constructions/structures; rather, only cross-serial dependencies defined on terminal strings have been discussed. Treating nested and cross-serial dependencies on a par may in fact mean that we are mixing apples and oranges, because nested dependency (as well as filler-gap/movement dependency and operator-variable dependency) is, as we have seen, a direct consequence of Merge (totally Merge-generable), whereas cross-serial dependency is a result of some interpretive mechanisms, with Merge only providing the necessary structural basis for the process (partially Merge-generable). We are of course aware that there are cross-serial dependency phenomena, typically in West Germanic languages [see Wurmbrand (2006) for an extensive review], reported and discussed in the literature. Although we cannot go into the details in this paper, and the definitive analyses of those phenomena surely remain to be properly formulated, it seems clear to us that those “cross-serial phenomena” can – and should – be naturally treated in terms of externalization mechanisms [see Huybregts (1984), Haegeman and Riemsdijk (1986), and subsequent works for much relevant discussion]. The generation of cross-serial dependencies, which requires the specification of linear (left-to-right) order, cannot be directly carried out by the core component of human language (Merge). Thus, those cross-serial phenomena, as well as, perhaps, the famous crossing case of “Affix Hopping” discussed in Chomsky (1957), ought to be handled in the externalization process.

Mainstream cognitive or neuroscientific investigations into human language have been centering around the Chomsky Hierarchy of weak generation. In their discussion, nested dependencies are treated as a hallmark of context-free grammars as distinguished from finite-state grammars, and cross-serial dependencies are used as testing grounds for context-sensitive grammars. However, these dependencies are usually characterized on the basis of terminal strings, and if we adopt the contemporary theory of Merge, we are instead led to an entirely different conception of the relevant dependencies. As we discussed above, nested dependencies naturally arise as a result of unbounded Merge, whereas cross-serial dependencies may appear in human language only insofar as the relevant structure is generated by Merge, and some other mechanisms/conditions are also fulfilled.

This conclusion may lead to an entirely new interpretation of the question of why nested dependencies abound, whereas cross-serial dependencies are severely limited in natural languages (see the discussion in section “Theoretical Background”). This is *not* because human language requires a characterization at the complexity of context-sensitive grammars or Turing machines (type-0 grammars), but because human language is so simple that it only avails itself of a minimal apparatus for strong generation, i.e., Merge. Merge-based phrase structures provide a direct basis for various nested dependencies, and also a rather partial (but necessary) means for characterizing limited kinds of cross-serial dependencies. In contrast, the human language faculty becomes rather extraneous whenever the task goes beyond the narrow channel of Merge-generability, such as when dealing with artificial nested/cross-serial dependences within terminal strings. Thus, dependencies defined on terminal strings are processed even if they are artificially imposed, but those processes are significantly enhanced when dependencies are Merge-generable. Ultimately, then, a weak generation of terminal strings is reduced to just a secondary effect of Merge-based strong generation. Consequently, our results also shed fresh light on another long-standing question, namely why the classical Chomsky Hierarchy does not constitute an entirely adequate scale along which human language is to be characterized and evaluated (cf., the notion of “mild” context-sensitivity). The Chomsky Hierarchy is typically a measure of weak generative capacity, and it is thus more or less orthogonal to the empirical study of human language (Chomsky, 1963, 1965).

Our conclusion should not be underestimated, since there are numerous studies that center on the relation between human language and the Chomsky Hierarchy. For instance, it has been reported that the computation of “regular-grammatical” dependencies of the form (AB)*^*n*^* (*n* = 2, 4) and “context-free” dependencies of the form A*^*n*^*B*^*n*^* (*n* = 2, 4) selectively activated different brain regions (the left frontal operculum and L. IFG, respectively) (Friederici et al., 2006). However, finite sequences of artificial symbols are a matter of weak generation at best, and there is little evidence that their participants were truly computing finite A*^*n*^*B*^*n*^* (where *n* = 2, 4) sequences in terms of hierarchical structures, i.e., phrase-structure grammars, let alone Merge-based human syntax. The literature on non-human animals’ capacities for computing finite (AB)*^*n*^* versus A*^*n*^*B*^*n*^* patterns are equally misguided (Gentner et al., 2006; Abe and Watanabe, 2011), if not only due to the unresolvable finiteness limitations.

We emphasize that the real novelty of our present experiments lies in its focus on Merge-generability, not merely phrase structures. Of course, there are quite a few neuroscientific studies that do try to discuss the relevance of phrase structures, but few have spoken to the Merge-generable versus non-Merge-generable distinctions [but see Ohta et al. (2013a, b) for notable exceptions]. For example, it has been reported that a selective cortical activation of the L. IFG for two-word phrase formation is enhanced compared to an unstructured list of two words (such as *this ship* vs. *stone, ship*) (Zaccarella and Friederici, 2015). This is a finding of importance, also consistent with our results, but the relevant dependencies between two adjacent words are so elementary that they may be characterized by *any* theory of strong generation. Another notable study provided an interesting set of data that support the primacy of structure-dependent computations in human language (Musso et al., 2003). Those authors asked German native speakers to learn two sets of transformational or pseudo-rules of Italian and Japanese (passive, negative construction, etc.). The first set of learned rules are real rules of the respective languages and thus consistent with the structure-dependence principle of human language, which holds that the applicability of transformational rules must be defined in terms of abstract phrase structures, not terminal strings. The second set of learned rules are unreal or artificially manipulated pseudo-rules that use the same lexicon as the respective languages but systematically violate the principle of structure-dependence, defined just on terminal strings (for example, putting the negation after the third word counting from the left). The results obtained in that study indicate that an increase of cortical activation in L. IFG was observed only for the acquisition of real structure-dependent rules, irrespective of the types of language. This work is significant in that it points to the primacy of phrase structures over terminal strings in the acquisition of transformational rules. It can thus be interpreted as constituting another empirical support for our broader hypothesis that abstract hierarchical structures generated by Merge are critical, not just for the formulation of transformational rules, but for possible dependencies in human language in general.

## Conclusion

In sum, our discussion points to the broad conclusion that all natural dependencies admissible in human language are Merge-generable, including certain types of nested, cross-serial, and transformational (such as filler-gap/movement) dependencies, and that non-Merge-generable dependencies of any type are extraneous to the human language faculty. There are only abstract hierarchical phrase structures in human language, generated all the way through via Merge. Here, we provided a novel set of neuroimaging data that confirm this general picture, thus corroborating the overarching hypothesis that human language at its core is a surprisingly simple system of unbounded Merge, and that Merge is the single generative engine underlying every aspect of linguistic computations.

## Data Availability Statement

The datasets generated for this study are available on request to the corresponding author.

## Ethics Statement

The studies involving human participants were reviewed and approved by the Institutional Review Board of The University of Tokyo, Komaba Campus. The patients/participants provided their written informed consent to participate in this study.

## Author Contributions

All authors contributed to the designing of the study and writing of the manuscript. KT, IN, SO, and KS conducted and analyzed the experiments.

## Conflict of Interest

The authors declare that the research was conducted in the absence of any commercial or financial relationships that could be construed as a potential conflict of interest.

## Footnotes

1 https://www.fil.ion.ucl.ac.uk/spm/

2 http://www.gin.cnrs.fr/en/tools/aal/

3 http://www.neuromorphometrics.com/
