# Supplementary material for: Merge-Generability as the Key Concept of Human Language: Evidence From Neuroscience
Source: Front Psychol. 2019 Nov 29;10:2673. doi: 10.3389/fpsyg.2019.02673 (PMC6895067; doi:10.3389/fpsyg.2019.02673)
Supplement: Supplementary file 1 [file Data_Sheet_1.pdf]

Natural (6W)

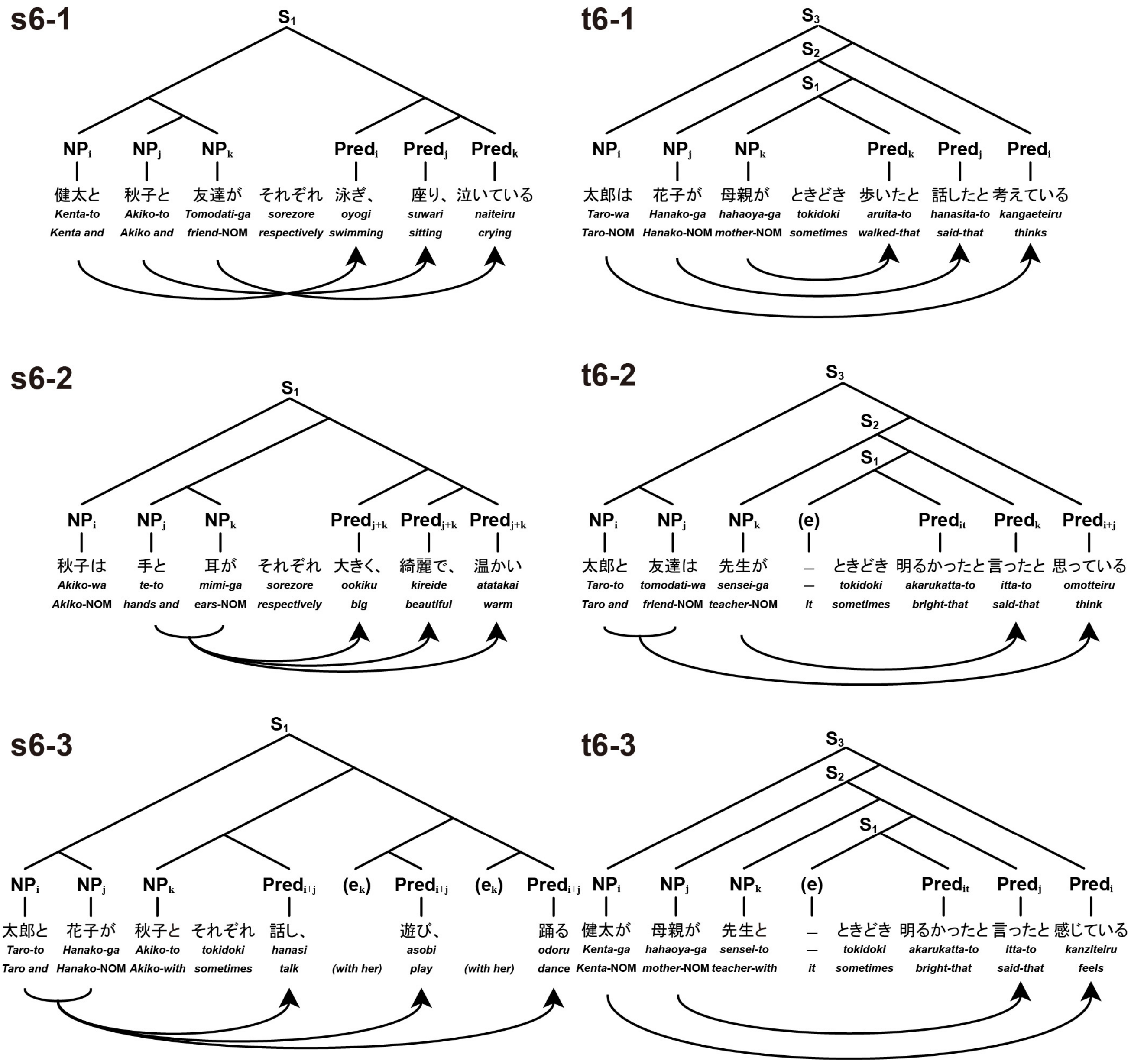

SUPPLEMENTARY FIGURE 1 Six types of Natural sentences with six words. Left: Three types of sentences with *sorezore*: *e.g.*, "*Kenta, Akiko, and* [*their*] *friend are swimming, sitting, and crying, respectively*" (s6-1), "*As for Akiko*, [*her*] *hands and ears are big, beautiful, and warm, respectively*" (s6-2), and "*Taro and Hanako talk with Akiko, play* [*with her*]*, and dance* [*with her*]*, respectively*" (s6-3). Right: Three types of sentences with *tokidoki*: *e.g.*, "*Taro thinks that Hanako said that* [*their*] *mother sometimes walked*" (t6-1), "*Taro and* [*his*] *friend think that* [*their*] *teacher said that it was sometimes bright*" (t6-2), and "*Kenta feels that* [*his*] *mother said with* [*his*] *teacher that it was sometimes bright*" (t6-3).

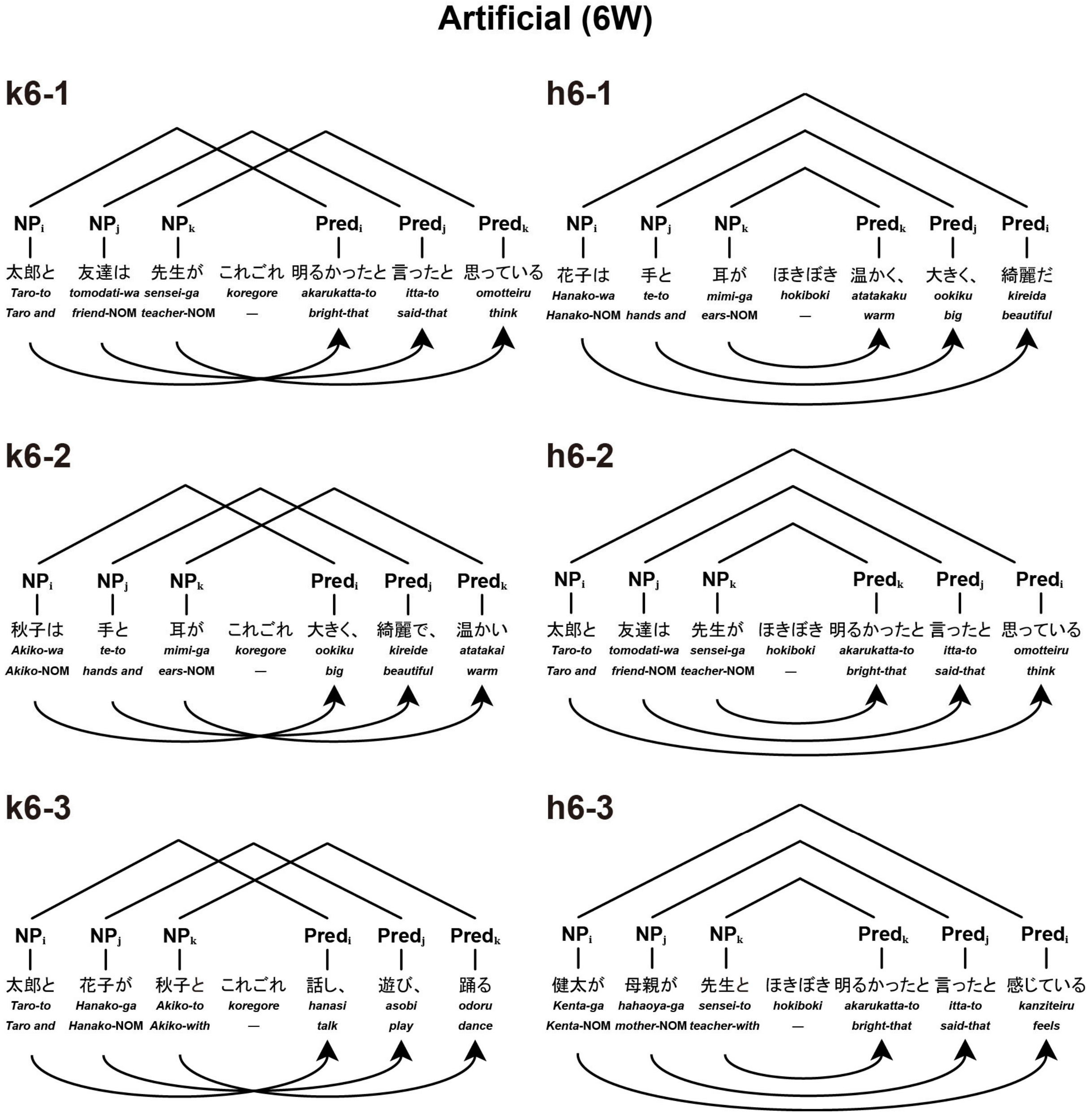

SUPPLEMENTARY FIGURE 2 Six types of Artificial sentences with six words. Left: artificial cross-serial dependencies (pairing relations between NPs and Preds). Right: artificial nested dependencies. In these examples, pseudowords (“*koregore*” or “*hokiboki*”) artificially forced the dependencies that are not Merge-generable.

APPENDIX

Task instructions

Day 1, first half

You will perform a task of judging *the correspondences between subjects and predicates* during MRI scanning.

While the stimuli are being shown, a small red cross will be presented at the center of the monitor.

Please look at the red cross, and do not move your eyes if possible.

During performing the task, please do not speak or read the sentences aloud.

・During the task, you will respond by pressing buttons.

・Please hold the controller with the button 1 on your left, and with the button 4 on your right.

・Please put your left thumb between the button 1 and 2, and put your right thumb between the button 3 and 4.

・Please press buttons with your thumbs as fast as you can.

・During scanning, you will wear goggles, and you will not be able to see the buttons.

・Please be careful not to overlook the sentences, and try to answer correctly as much as possible.

・*If a sentence disappears before you respond, please do not try to press buttons anymore, and concentrate on the next sentence.*

・Japanese sentences are shown on the monitor, in the order of subjects, an adverb, and predicates.

・*Please read the sentences carefully, and do not overlook particles such as* -wa, -ga *and* -to.

・After a sentence disappears, one predicate will be presented in the upper row, and three subjects will be presented in the lower row. Some words which are not contained in the sentence may appear in the alternatives.

・From the three alternatives, choose *in the original sentence the most appropriate subject, which corresponds to that predicate*.

・The alternatives of subjects from left to right correspond to the buttons 1, 2, and 3, respectively.

♦ **Sorezore (4W)**

taro-to *hanako-ga* *sorezore* *hasiri,* *suwatteiru*

[*Taro and* *Hanako*-NOM *respectively* *running* *sitting*]

(Taro and Hanako are running and sitting, respectively)

[NB: Square brackets denote additional information to the reader.]

[A predicate and alternatives:]

hasiru

taro *hanako* *taro-to-hanako*

1 2 3

○ × × (Use only leftmost three buttons)

taro-ga *teasi-ga* *sorezore* *hosoku,* *siroi*

[*Taro*-NOM *limbs*-NOM *respectively* *skinny* *white*]

(Limbs are skinny and white, respectively)

oyako-ga *taro-to* *sorezore* *odori,* *asobu*

[*parent-and-child*-NOM *Taro-with* *respectively* *dance* *play*]

(Parent-and-child dance and play, respectively)

Oyako [parent-and-child] will not be presented as separate alternatives, i.e., *oya* (parent) and *ko* (child), but it will be always presented as one alternative; the same holds for *ryosin* [parents], *senseigata* [teachers], *watasitachi* [we], *simai* [sisters], *sohubo* [grandparents], and *husai* [husband-and wife]. [NB: The second NP for this sentence type was not used as an alternative.]

♦ **Tokidoki (4W)**

taro-ga *hanako-ga* *tokidoki* *aruita-to* *hanasiteiru*

[*Taro*-NOM *Hanako*-NOM *sometimes* *walked-that* *says*]

(Hanako walked, and Taro says)

taro-to *hanako-ga* *tokidoki* *akarukattato* *hanasiteiru*

[*Taro and* *Hanako*-NOM *sometimes* *bright-that* *say*]

(Taro and Hanako say)

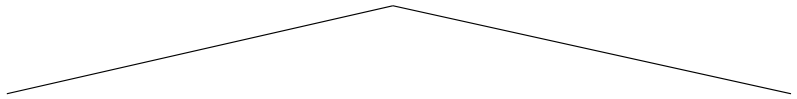

taro-ga	*hanako-to*	*tokidoki*	*akarukatta-to*	*hanasiteiru*
[*Taro*-NOM	*Hanako-with*	*sometimes*	*bright-that*	*says*]

(Taro says)

[NB: The second NP for this sentence type was not used as an alternative.]

Practice: You will practice the task until you can perform two serial sets of trials, each with at least four correct answers in six trials.

In the MRI scanner, you will have 8 runs, and each run contains 19 trials.

Day 1, second half

・*Please read the sentences carefully, and do not overlook particles such as* -wa, -ga *and* -to.

・After a sentence disappears, one predicate will be presented in the upper row, and four subjects will be presented in the lower row. Some words which are not contained in the sentence may appear in the alternatives.

・From the four alternatives, choose *in the original sentence the most appropriate subject, which corresponds to that predicate*.

♦ Sorezore (6W)

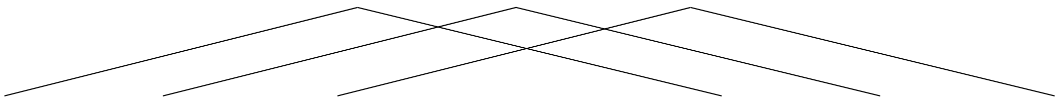

taro-to	*hanako-to*	*titioya-ga*	*sorezore*	*hasiri*,	*suwari*,	*waratteiru*
[*Taro and*	*Hanako and*	*father*-NOM	*respectively*	*running*	*sitting*	*laughing*]

[A predicate and alternatives:]

		warau	
taro	*hanako*	*titioya*	*taro-to-hanako*
1	2	3	4
×	×	○	×

taro-wa	*te-to*	*ashi-ga*	*sorezore*	*hosoku,*	*siroku,*	*atatakai*
[*Taro*-NOM	*hands and*	*legs*-NOM	*respectively*	*skinny*	*white*	*warm*]

titioya-to	*hahaoya-ga*	*taro-to*	*sorezore*	*ai,*	*hanasi,*	*odoru*
[*father and*	*mother*-NOM	*Taro-with*	*respectively*	*meet*	*talk*	*dance*]

♦ Tokidoki (6W)

taro-wa	*hanako-ga*	*titioya-ga*	*tokidoki*	*aruita-to*	*omotta-to*	*hanasiteiru*
[*Taro*-NOM	*Hanako*-NOM	*father*-NOM	*sometimes*	*walked-that*	*though-that*	*says*]

taro-to *titioya-wa* *hahaoya-ga* *tokidoki* *akarukatta-to* *hanasita-to* *kangaeteiru*

[*Taro and* *father*-NOM *mother*-NOM *sometimes* *bright-that* *said-that* *think*]

taro-ga *hahaoya-ga* *titioya-to* *tokidoki* *akarukatta-to* *hanasita-to* *kangaeteiru*

[*Taro*-NOM *Hanako*-NOM *father-with* *sometimes* *bright-that* *said-that* *think*]

Practice: You will practice the task until you can perform two serial sets of trials, each with at least four correct answers in six trials.

In the MRI scanner, you will have 9 runs, and each run contains 13 trials.

Day 2, first half

A task of judging the correspondences between words

・In this task, novel words of *koregore* and *hokiboki* will be used.

・According to these novel words, *correspondences between other four words are determined as shown by the diagrams below.* These novel words work completely differently from *sorezore* and *tokidoki* used on Day 1.

・*Under any sentence types, please remember the correspondences between words, not the meaning of a sentence.*

・From the three alternatives, choose one corresponding to that word in the upper row. Some words which are not contained in the sentence may appear in the alternatives.

♦ **Koregore (4W)**

taro-to *hanako-ga* *koregore* *akarukatta-to* *hanasiteiru*

[*Taro and* *Hanako*-NOM — *bright-that* *say*]

[A predicate and alternatives:]

	hanasu	
taro	*hanako*	*kenta*
1	2	3
○	×	×

(Use only leftmost three buttons)

taro-ga *teasi-ga* *koregore* *hosoku,* *siroi*

[*Taro*-NOM *limbs*-NOM — *skinny* *white*]

oyako-ga *taro-to* *koregore* *odori,* *asobu*

[*parent-and-child*-NOM *Taro-with* — *dance* *play*]

♦ **Hokiboki (4W)**

taro-to *hanako-ga* *hokiboki* *akarukatta-to* *hanasiteiru*

[*Taro and* *Hanako*-NOM — *brigh-that* *say*]

taro-ga *hanako-to* *hokiboki* *kurakatta-to* *kanjiteiru*

[*Taro*-NOM *Hanako-with* — *dark-that* *feels*]

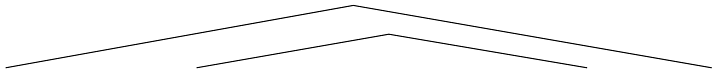

taro-ga	*teasi-ga*	*hokiboki*	*hosoku,*	*siroi*
[*Taro*-NOM	*limbs*-NOM	—	*skinny*	*white*]

Practice: You will practice the task until you can perform two serial sets of trials, each with at least four correct answers in six trials.

In the MRI scanner, you will have 8 runs, and each run contains 19 trials.

Day 2, second half

Just like the first half, you will perform the task of judging the correspondences between six words in the second half.

♦ Koregore (6W)

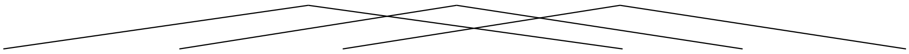

titioya-to	*hahaoya-ga*	*taro-to*	*koregore*	*ai,*	*hanasi,*	*odoru*
[*father and*	*mother*-NOM	*Taro-with*	—	*meet*	*talk*	*dance*]

[A predicate and alternatives:]

	au		
titioya	*hahaoya*	*taro*	*kenta*
1	2	3	4
○	×	×	×

taro-wa	*te-to*	*asi-ga*	*koregore*	*hosoku,*	*siroku,*	*atatakai*
[*Taro*-NOM	*hands and*	*legs*-NOM	—	*skinny*	*white*	*warm*]

taro-to	*titioya-wa*	*hahaoya-ga*	*koregore*	*akarukatta-to*	*hanasita-to*	*kangaeteiru*
[*Taro and*	*father*-NOM	*mother*-NOM	—	*bright-that*	*said-that*	*think*]

♦ **Hokiboki (6W)**

taro-to	*titioya-wa*	*hahaoya-ga*	*hokiboki*	*akarukatta-to*	*hanasita-to*	*kangaeteiru*
[*Taro and*	*father*-NOM	*mother*-NOM	—	*bright-that*	*said-that*	*think*]

taro-to	*titioya-wa*	*hahaoya-ga*	*hokiboki*	*kurakatta-to*	*itta-to*	*kanjiteiru*
[*Taro and*	*father*-NOM	*mother*-NOM	—	*dark- that*	*said that*	*feel*]

taro-wa	*te-to*	*asi-ga*	*hokiboki*	*hosoku,*	*siroku,*	*atatakai*
[*Taro*-NOM	*hands and*	*legs*-NOM	—	*skinny*	*white*	*warm*]

Practice: You will practice the task until you can perform two serial sets of trials, each with at least four correct answers in six trials.

In the MRI scanner, you will have 8 runs, and each run contains 19 trials.
